# What Makes Umbilical Cord Tissue-Derived Mesenchymal Stromal Cells Superior Immunomodulators When Compared to Bone Marrow Derived Mesenchymal Stromal Cells?

**DOI:** 10.1155/2015/583984

**Published:** 2015-05-12

**Authors:** R. N. Bárcia, J. M. Santos, M. Filipe, M. Teixeira, J. P. Martins, J. Almeida, A. Água-Doce, S. C. P. Almeida, A. Varela, S. Pohl, K. E. J. Dittmar, S. Calado, S. I. Simões, M. M. Gaspar, M. E. M. Cruz, W. Lindenmaier, L. Graça, H. Cruz, P. E. Cruz

**Affiliations:** ^1^ECBio S.A. - R&D in Biotechnology, Rua Henrique Paiva Couceiro, 27, 2700-451 Amadora, Portugal; ^2^Instituto de Medicina Molecular, Faculdade de Medicina da Universidade de Lisboa, Avenida Professor Egas Moniz, 1649-028 Lisboa, Portugal; ^3^Helmholtz Centre for Infection Research, Department of Gene Regulation and Differentiation, Inhoffenstaße 7, 38124 Braunschweig, Germany; ^4^Research Institute for Medicines (iMed.ULisboa), Faculty of Pharmacy, University of Lisbon, Avenida Professor Gama Pinto, 1649-003 Lisbon, Portugal

## Abstract

MSCs derived from the umbilical cord tissue, termed UCX, were investigated for their immunomodulatory properties and compared to bone marrow-derived MSCs (BM-MSCs), the gold-standard in immunotherapy. Immunogenicity and immunosuppression were assessed by mixed lymphocyte reactions, suppression of lymphocyte proliferation and induction of regulatory T cells. Results showed that UCX were less immunogenic and showed higher immunosuppression activity than BM-MSCs. Further, UCX did not need prior activation or priming to exert their immunomodulatory effects. This was further corroborated *in vivo* in a model of acute inflammation. To elucidate the potency differences observed between UCX and BM-MSCs, gene expression related to immune modulation was analysed in both cell types. Several gene expression profile differences were found between UCX and BM-MSCs, namely decreased expression of *HLA-DRA*, *HO-1*, *IGFBP1*, 4 and 6, *ILR1*, *IL6R* and *PTGES* and increased expression of *CD200*, *CD273*, *CD274*, *IL1B*, *IL-8*, *LIF* and *TGFB2*. The latter were confirmed at the protein expression level. Overall, these results show that UCX seem to be naturally more potent immunosuppressors and less immunogenic than BM-MSCs. We propose that these differences may be due to increased levels of immunomodulatory surface proteins such as CD200, CD273, CD274 and cytokines such as IL1*β*, IL-8, LIF and TGF*β*2.

## 1. Introduction

Mesenchymal stromal cells (MSCs) from a variety of sources have been described as having interesting immunomodulatory characteristics and have thus been studied in the context of immunotherapy. Lack of expression of MHC II molecules and low expression of MHC I molecules, as well as low level expression of costimulatory molecules such as CD80 and CD86 [1], all contribute to the low immunogenicity observed in these cells and render them easily accepted in an allogeneic noncompatible donor setting. Further, MSCs have demonstrated to have other properties that enable their use in allogeneic transplantation and in immunotherapy [2]. They have been shown to suppress T cell activation by protecting quiescent T cells from death, to arrest T cells in G0/G1 phase of cell cycle, and to promote apoptosis of activated T cells [3]. MSCs have also been shown to inhibit B cell proliferation and differentiation [4, 5], the proliferation of natural killer (NK) cells, and their cytokine production [6, 7] and to inhibit the ability of dendritic cells to stimulate alloresponses [8–10]. In addition, MSCs are thought to affect peripheral tolerance by inducing Tregs [11, 12]. Many mechanisms have been proposed for the immunomodulatory effects of MSCs [13]. It is widely accepted that this immunomodulation is exerted mainly through a paracrine effect, via the secretion of soluble factors such as transforming growth factor-*β*1 (TGF-*β*) and hepatocyte growth factor (HGF) [14], soluble human leukocyte antigen-G (HLA-G) [12, 15], interleukin-10 (IL10) [16, 17], prostaglandin E_2_ (PGE_2_), nitric oxide (NO) [18], galectin-1 [19] and galectin-3 [20], interleukin-6 (IL-6) [9], indoleamine 2,3-dioxygenase (IDO) [21], leukaemia inhibitory factor (LIF) [22, 23], heme oxygenase-1 (HO-1) [24], chemokine (C-C motif) ligand 2 (CCL2) [25], and insulin-like growth factor-binding proteins (IGFBP) [26]. Beyond soluble factors, CD200, CD276, and HLA-E have recently been suggested to be involved in the immunoregulatory mechanisms of MSCs [27, 28]. Together, these mechanisms also contribute to the anti-inflammatory effect of MSCs [29] and, when coupled with their ability to home to inflammation sites, make MSCs a powerful therapeutic agent for autoimmune and inflammatory disorders.

UCX are isolated from the umbilical cord tissue (or Wharton's jelly) according to proprietary technology [30]. It is an extremely robust method and reproducible one that has proved to be adequate for stem cell banking. It generates high yields of cells with almost 100% success rate and very few microbial contaminations. UCX are highly expandable and can be safely cultured up to 50 generations without losing genomic stability and their most relevant therapeutic capabilities.

With the intent of taking UCX into the clinic, the method of isolation, expansion, and cryopreservation of UCX has been adapted in order to generate an Advanced Therapy Medicinal Product (ATMP) [31]. The aim of this work is to study the characteristics of UCX, particularly with regard to immunogenicity and immune regulation, and compare them to BM-MSCs, the current gold-standard in stem cell-based immunotherapy.

## 2. Materials and Methods

### 2.1. Cells and Reagents

#### 2.1.1. UCX

Umbilical cord donations were obtained from local hospitals and clinics with approved ethics committee and written informed consents, according to Directive 2004/23/EC of the European Parliament (Portuguese Law 22/2007 of June 29) and the Declaration of Helsinki.

Human umbilical cord tissue-derived MSCs were isolated according to Santos et al. [30] and were herein named UCX. In brief, cords were depleted of blood, transported to the laboratory facilities, and processed within a period up to 72 hours after collection. The umbilical cords were immersed in a decontaminating solution of HBSS (1×) (Sigma-Aldrich, St. Louis, MO, USA) supplemented with additional 1 g/L glucose (Sigma-Aldrich, St. Louis, MO, USA), 1 mg/L ciprofloxacin-HCl (Santa Cruz Biotechnology, Heidelberg, Germany), and antibiotic-antimycotic solution (1×) (Gibco, Madrid, Spain), overnight, 4°C prior to processing. The cords were washed, sectioned, and digested with a mixture of collagenase, type II (Sigma-Aldrich, St. Louis, MO, USA), and porcine trypsin (Sigma-Aldrich, St. Louis, MO, USA) using a constant ratio between tissue mass (g), bottom surface area of the digestion container (cm^2^), digestion solution volume (mL), and the total flask volume (mL). Cells dissociated from the tissue during digestion which were able to adhere to the surface of a culture flask (EasyFlasks; Nunc, Schnelldorf, Germany) during a static 30 min horizontal incubation period in static monolayer culture at 37°C in a humidified atmosphere containing 7% CO_2_ in basal MSC culture medium (*α*-MEM basal medium with 1 g/L glucose and 2 mM glutamine (Sigma-Aldrich, St. Louis, MO, USA) supplemented with 20% foetal bovine serum (FBS) (Gibco, Madrid, Spain)). Nonadherent cells were removed the next day and fresh medium was added. Cultures were maintained and complete exchange of culture medium was performed twice weekly. Fibroblast-like colonies were observed regularly and recovered when confluence was observed.

Cells were washed with PBS and then detached using 0.25% (w/v) trypsin-EDTA (Sigma-Aldrich, St. Louis, MO, USA). UCX were seeded at 5000–10000 cells/cm^2^ on culture flasks (Nunc, Schnelldorf, Germany) in MSC medium. Cells were incubated at 37°C and 7% CO_2_ in a humidified incubator and fed by replacing the culture medium twice weekly until confluence was observed.

#### 2.1.2. Bone Marrow MSCs

Freshly isolated MSCs from the bone marrow do not proliferate well beyond passage 8 and thus cell quantities are limited. For that reason, commercially available BM-MSCs (Innoprot, Vizcaya, Spain) were used since these cells seem to have a higher proliferative capacity than MSCs freshly isolated from the BM and are capable of growing until at least passage 15 (as guaranteed by the vendor). BM-MSCs were thawed and grown as per supplier's instructions in the same media used for UCX. Cells were used between passages 4 and 8. To note, bone and bone marrow derived mesenchymal stromal cell lines G3 and M7 used on the Affymetrix gene array have been described [32]. In addition, in order to increase the number of samples used, BM-MSCs derived from individuals were obtained from the Lobato da Silva Lab, Technical University of Lisbon [33], and used between passages 5 and 8 in the experiments where protein expression of CD200, CD273, CD274, IL-1*β*, IL-8, LIF, and TGF-*β*2 was studied.

#### 2.1.3. Molt4 Cells

Molt4 cells, a human acute lymphoblastic leukaemia T cell line (Minowada, 1972, PMCID: PMC1553679), were used as controls and were grown in RPMI 1640 medium supplemented with 2 mM L-glutamine and 10% foetal bovine serum.

### 2.2. Cell Priming and Conditioned Media

For priming, cells were seeded and cultured in reduced FBS (5%) until 90% confluence was observed. Cells were washed with *α*-MEM basal medium (Sigma-Aldrich, St. Louis, MO, USA). 10 ng/mL TNF*α* (Jena Biosciences, Jena, Germany) and 10 ng/mL INF*γ* were added to cells. For controls, no factors were added. Cells were incubated at 37°C in a humidified atmosphere containing 7% CO_2_ for 24 hours. Cells were either (1) detached, as previously described, for the collection of the primed cells or (2) incubated for an additional 48 hours for the production of conditioned medium (CM) in fresh *α*-MEM basal medium. Both CM of primed cells and controls were concentrated in 5 kDa cut-off spin concentrators (Agilent Technologies, Santa Clara, CA, USA) as per manufacturer's recommendations and relevant proteins quantified by ELISA (RayBiotech, Norcross, GA, USA) as per manufacturer's instructions.

### 2.3. Immunophenotyping

To analyse cell-surface expression, cells were detached, counted, and labelled with the following anti-human antibodies: CD14-PerCp/Cy5.5; CD19-Pacific Blue; CD31-FITC; CD34-FITC; CD44-APC; CD45-PerCp/Cy5.5; CD73-APC; CD90-PE and HLA-DR-Pacific Blue, CD200-Alexa Fluor 647, CD273-PE and CD274-PE all from Biolegend (San Diego, CA, USA), and also CD105-PE (eBioscience, San Diego, CA, USA). The mouse isotype antibodies used as the respective controls were Pacific Blue IgG1; Pacific Blue IgG2a; IgG1k PerCp/Cy5.5; IgG2a PerCp/Cy5.5; IgG1k PE; IgG1k APC and IgG1k FITC, Alexa Fluor 647 IgG1k, and PE IgG1k all from Biolegend (San Diego, CA, USA). 10 000 labelled cells were acquired using a Gallios Flow cytometer (Beckman Coulter, Brea, CA, USA) and analysed with Kaluza software (Beckman Coulter, Brea, CA, USA).

### 2.4. Quantification of Secreted Factors

The secretion of cytokines produced by UCX and BM-MSC cells and secreted into the culture medium was assessed by quantifying LIF, TGF-*β*2, and IL-1*β* in the appropriate conditioned media. Commercially available ELISA kits were used (R&D Systems, Minneapolis, MN, USA), according to the manufacturer's instructions. The quantification of IL-8 was performed using a commercially available kit (FlowCytomix; eBioscience, San Diego, CA, USA). Samples from conditioned media of UCX and BM-MSC were used and the protocol followed according to manufacturer's instructions. Samples were acquired on a Gallios imaging flow cytometer (Beckman Coulter, Brea, CA, USA) and the results were obtained using FlowCytomix Pro 3.0 Software.

### 2.5. Trilineage Differentiation

Adipogenic differentiation was induced by cyclic changes of induction and maintenance media in cells cultivated after confluency as previously described [34]. After three cycles of media changes, adipogenic differentiation was apparent by intracellular accumulation of lipid-rich vacuoles stained with Oil Red O.

To promote chondrogenic differentiation, cell pellets were prepared and cultured for 3 weeks in complete chondrogenic differentiation medium, as previously described [34]. After the culture period, fixed, deparaffinized, and rehydrated sections were stained with 1% (w/v) alcian blue (Sigma-Aldrich) in 3% (v/v) acetic acid (Sigma-Aldrich) and bright blue stained glycosaminoglycans and mucopolysaccharides were visible.

Osteogenic induction medium was used to promote differentiation as previously described [34]. The onset of osteoblast formation was evaluated after 4 weeks by the detection of alkaline phosphatase activity using the leukocyte alkaline phosphatase kit (Sigma-Aldrich) according to the manufacturer's protocol.

### 2.6. Mixed Lymphocyte Reactions

Peripheral blood from healthy volunteers was collected with informed consent in heparin, diluted 1 : 1 (v/v) with PBS 1× and mixed with half the volume of Histopaque-1077 (Sigma-Aldrich, St. Louis, MO, USA). The mixed lymphocyte reaction (MLR) was performed in 96-well microtiter plates using RPMI (Gibco, Madrid, Spain) and 5% human serum obtained from the specific donor. Peripheral blood mononuclear cells (PBMCs) were obtained and cultured at 2 × 10^5^ cells per well. Stimulator cells were irradiated with 50 Gy (Gammacell ELAN 3000, Best Theratronics, Ontario, Canada) and added to the culture at 20 000 cells per well, resulting in a 1 : 10 MSC : PBMC ratio. Quadruplicate cultures were performed for each condition. Cultures were incubated at 37°C in 5% CO_2_ for 6 days, pulsed with [^3^H]thymidine (1 microCi per well, Amersham Biosciences, Piscataway, NJ, USA) for 16 hours, and the cells were harvested onto filter mats using a Tomtec 96-well cell harvester (Perkin Elmer, Waltham, MA, USA). Radioactivity incorporated into the dividing cells was determined using a scintillation counter (Microbeta Trilux Scintillation and Luminescence Counter 145 LSC, Perkin Elmer, Waltham, MA, USA).

### 2.7. Immunosuppression and Induction of Treg Conversion

For immune suppression assays, PBMCs collected after Ficoll gradient were cultured at 2 × 10^5^ cells per well cultured in RPMI (Gibco) supplemented with 5% HEPES (Gibco), 5% Pen-Strep (Gibco), 5% NaPyr (Gibco), and 5% human serum obtained from the specific donor and were stimulated with anti-CD3 (eBioscience), anti-CD28 (eBioscience), and IL-2 (eBioscience). Suppressor cells (MSCs and non-MSC controls) were irradiated with 50 Gy (Gammacell ELAN 3000, Best Theratronics) prior to addition to the culture at 20 000 cells per well, resulting in a 1 : 10 MSC : PBMC ratio. Quadruplicate cultures were performed for each condition. Cultures were incubated at 37°C in 5% CO_2_ for 6 days, pulsed with [^3^H]thymidine (1 microCi per well, Amersham Biosciences, Piscataway) for 16 hours, and the cells were harvested onto filter mats using a Tomtec 96-well cell harvester (Perkin Elmer). Radioactivity incorporated into the dividing cells was determined using a scintillation counter (Microbeta Trilux Scintillation and Luminescence Counter 145 LSC, Perkin Elmer).

For the induction of Tregs, PBMCs were collected from the Ficoll gradient after centrifugation at 720 g for 30′ at RT, washed with PBS containing 2% FCS, and then stained with mAbs against human CD3, CD4, and CD25 (eBioscience, San Diego, CA, USA) for cell sorting. The purified CD3^+^CD4^+^CD25^−^ T cells were cultured in plate-bound *α*huCD3 (2.5 *μ*g/mL, eBioscience, San Diego, CA, USA) in 96-well flat-bottom plates in the following conditions. Briefly, 1 × 10^5^ purified T cells/well were cultured in the presence of *α*huCD28 (2 *μ*g/mL, eBioscience, San Diego, CA, USA), huIL-2 (20 U/mL, Peprotech, London, UK), and TGF-*β* (10 ng/mL, R&D Systems, Abingdon, UK) or the indicated cell lines (irradiated as described), in replacement of TGF-*β*, in a ratio of 1 : 1 to the T cells. All conditions were performed in triplicate wells. After 5 days in culture at 37°C with 5% CO_2_, cells were stained with mAbs against human CD3, CD4, and CD25 (eBioscience, San Diego, CA, USA) and then stained for huFoxp3 as described by the manufacturer (eBioscience, San Diego, CA, USA). The analysis was performed on the converted CD3^+^CD4^+^ CD25^+^Foxp3^+^ regulatory T cells.

The immunosuppressive studies (T cell suppression and Treg induction) were performed in at least 2 independent experiments. Each graph in Figures 3(a) and 3(b) is representative of 1 experiment, where 1 donor for each MSC source was used.

### 2.8. Acute Carrageenan-Induced Arthritic (CIA) Inflammatory Model

All animal experiments were carried out with the permission of the local Animal Ethical Committee in accordance with the EU Directive (2010/63/UE), Portuguese law (DR 129/92, Portaria 1005/92), and all the applicable legislation. All animals were obtained from Charles River Laboratories (Santa Perpetua de Mogoda, Spain) and kept under standard laboratory conditions. Carrageenan was purchased from Sigma-Aldrich (St. Louis, MO, USA). Wistar rats, aged 7 to 8 weeks, were used. Paw oedema was induced by intradermal injection of 0.1 mL of a 1% carrageenan saline solution into the subplantar area of the right hind paw [35]. The evaluation of the paw oedema was monitored by changes of the volume of right and left paws by a water displacement method, using a plethysmometer (Ugo Basile, Comerio, Italy). The paws were immersed in the measurement cell up to the hair line to the ankle to determine the immersed organ volume in mL. Measurements were made immediately before the injection of carrageenan and thereafter at 2 hr intervals for 6 hr. Oedema was expressed as the increase in paw volume (milliliters) after carrageenan injection relative to the preinjection value for each animal. Cells at a concentration of 1.7 × 10^6^ in a total volume of 0.1 mL were administered by intra-articular (i.a.) injection in the right paw, 1 hr before carrageenan injection.

### 2.9. Gene Expression Profiling

RNA from UCX cultivated in a MEM with the supplements indicated and from BM-MSCs cultivated in DMEM supplemented with 10% FBS was isolated using the RNeasy Mini Kit (Qiagen, Hilden, Germany), following the protocols of the manufacturer. RNA was isolated using the RNeasy Mini Kit (Qiagen, Hilden, Germany), following the protocols of the manufacturer. About 5 × 10^6^ cells were collected after trypsinization. After cell lysis, homogenization was performed by passing the lysate 5 times through a 20-gauge syringe and DNAse digestion was used to eliminate DNA contamination. Quality and integrity of the total RNA isolated were controlled on a bioanalyzer (Agilent Technologies; Waldbronn, Germany).

5 *μ*g of total RNA was used for biotinylated target synthesis according to standard protocols supplied by the manufacturer (Affymetrix; Santa Clara, CA). Briefly, RNA was converted to dsDNA using 100 pmol of a T7T23V primer (Eurogentec; Seraing, Belgium) containing a T7 promoter. The cDNA was then used directly in an* in vitro* transcription reaction in the presence of biotinylated nucleotides. The concentration of biotin-labelled cRNA was determined by UV absorbance. For hybridization, 10 *μ*g of each biotinylated cRNA preparation was fragmented and placed in a hybridization cocktail containing also 4 biotinylated hybridization controls (BioB, BioC, BioD, and Cre) as recommended by the manufacturer. Samples were hybridized for 16 hours to Affymetrix Gene Chip HG_U133 Plus 2.0, representing about 47000 human transcripts. After hybridization, the GeneChips were washed, stained with SA-PE, and read using an Affymetrix GeneChip fluidic station and scanner.

The resulting dataset is available under Gene Expression Omnibus (GEO) Accession number GSE51869. Analysis of microarray data was performed using the Affymetrix Microarray Suite 5.0 and BRB Array Tools 4.2. All array experiments were normalized using RMA.

The differences in relative expression between UCX and BM-MCS were calculated as a percentage of BM-MSCs expression. Furthermore, only genes where this difference was statistically significant were included.

## 3. Results

### 3.1. Characterization of MSCs from Both Sources

**Figure 1:**
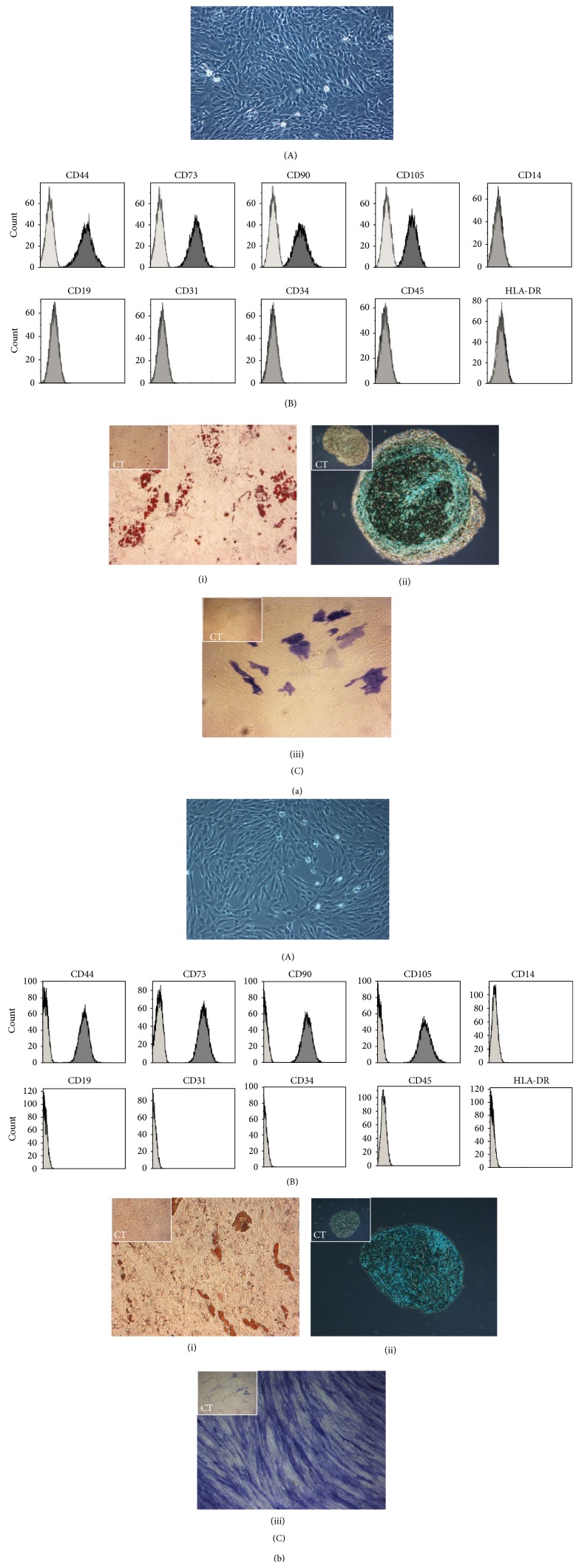
Characterization of UCX (a) and BM-MSCs (b). (A) Both MSCs showed a fibroblast-like morphology (175x amplified). Representative photo of cultured cells is at passage 5, 3 days after seeding 10.000 cells/cm^2^. (B) Flow cytometry analysis of MSC surface-markers expression of cells expanded for 5 passages. (C) Both MSCs were capable of trilineage differentiation. After 21 days of exposure to adipogenic, chondrogenic, and osteogenic differentiation media, results showed (i) Oil Red O staining for adipocytes, (ii) alcian blue staining for chondrocytes, and (iii) alkaline phosphatase staining for osteoblasts. Controls (CT) are shown in the upper left corner of each differentiation photo.

Both MSCs entirely fulfil the MSC criteria as defined by the International Society for Cellular Therapy (ISCT) [36]. Cells were adherent to plastic showing a fibroblast-like morphology and were positive for CD105, CD90, and CD73 and positive for CD19, CD34, CD45, CD14, and HLA-DR (Figures 1(a) and 1(b)). Expression of CD31 and CD44 was also analysed as it is relevant given the origin of UCX. The residual expression of CD31 (<2%) ruled out any significant contamination of the UCX population with endothelial cells [37] while high CD44 expression (>95%) confirmed the intrinsic capacity of UCX to participate in MSC-like cellular functions, such as lymphocyte activation, recirculation, and homing and their stromal origin [38]. Both MSCs demonstrated the ability to differentiate into adipocytes, chondrocytes, and osteoblasts-like cells (Figure 1(C)), though, as expected, BM-MSCs showed an increased capacity for osteogenic differentiation, whereas UCX showed capacity for chondrogenic differentiation.

### 3.2. UCX Are Less Immunogenic Than BM-MSCs

**Figure 2:**
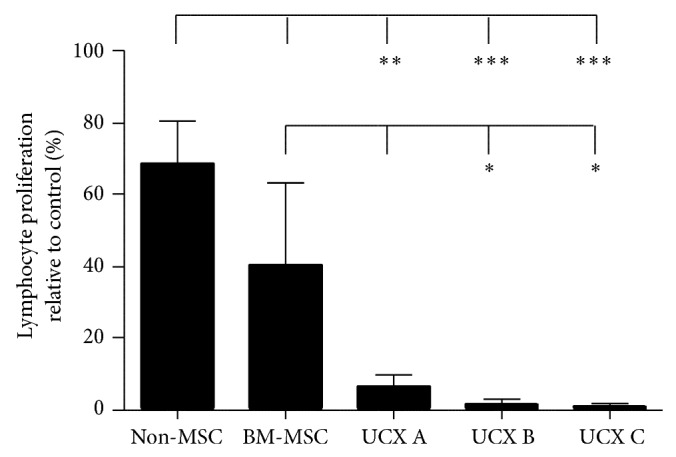
UCX are less immunogenic than BM-MSCs. PBMCs from two different donors were incubated with a non-MSC cell line (Molt4), BM-MSC, and UCX from 3 independent donors. Lymphocyte proliferation was measured as [^3^H]thymidine uptake (ccpm). UCX, and to a lesser extent BM-MSC, failed to induce lymphocyte activation in an allogeneic MLR. Values are represented as mean ± s.e.m. and statistically significant differences are indicated by asterisks (one-way ANOVA with a Tukey posttest; ^∗^ *P* < 0.05, ^∗∗^ *P* < 0.01, and ^∗∗∗^ *P* < 0.001).

A mixed lymphocyte reaction (MLR) was performed (Figure 2) and immunogenicity was measured by the level of proliferation of the PBMCs when cocultured with irradiated cells. The immunogenic potential of UCX derived from 3 individual umbilical cords (UCX A, UCX B, and UCX C) was compared with BM-MSCs and a non-MSCs human cell line (Molt4), as a control. Results were expressed as percentage of lymphocyte proliferation relative to proliferation observed when PBMCs from one donor were incubated with PBMCs from the other donor. The percentage of lymphocyte proliferation in culture with irradiated non-MSCs was about 70% (Figure 2). MSCs showed a clear reduced ability to induce lymphocyte proliferation corroborating their known low immunogenic potential. Interestingly, UCX from different donors consistently resulted in lower lymphocyte proliferation (1–12%) when compared to BM-MSCs (30–40%). This data indicates that UCX are less immunogenic than BM-MSC.

### 3.3. UCX Are More Immunosuppressive Than BM-MSCs

**Figure 3:**
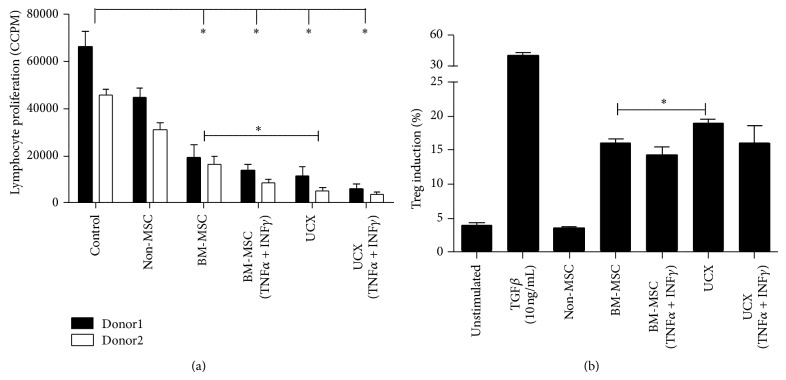
UCX are more potent immunosuppressors than BM-MSCs. (a) Activation of PBMCs from two different donors was induced by incubation with anti-CD3, anti-CD28, and IL-2 (control). Suppression of lymphocyte proliferation was analysed in the presence of irradiated non-MSCs (Molt4), BM-MSCs, and UCX (both naïve and primed with 10 ng/mL of TNF*α* and IFN*γ*). Independent of donor, lymphocyte proliferation was significantly suppressed when cells were cocultured with MSCs, but not with non-MSCs. Values are represented as mean ± s.e.m. and statistically significant differences are indicated by asterisks (nonparametric *t*-test Mann Whitney, ^∗^ *P* < 0.05). (b) Treg conversion was assayed by using FACS-sorted CD3^+^CD4^+^CD25^−^ T cells that were activated with anti-CD3, anti-CD28, and IL-2 alone and with the addition of TGF-*β*. The effect of irradiated UCX and BM-MSCs in T cell conversion was analysed in cultures without exogenous TGF-*β* by flow cytometry analysis of CD25 and Foxp3 expression. Values are represented as mean ± s.e.m. and statistically significant differences are indicated by asterisks (unpaired *t*-test, ^∗^ *P* < 0.05).

*In vitro* analysis was performed to functionally test immunosuppressive properties of both types of MSCs. This time, PBMCs from two donors were activated by incubation with anti-CD3, anti-CD28, and interleukin-2 (IL-2) (Figure 3(a), control).* In vitro* immunosuppression was assessed by measuring [^3^H]thymidine uptake (ccpm) of the proliferating PBMCs in the presence or absence of MSCs or a non-MSC control (Molt4). Since it has been suggested that MSCs demonstrate an enhanced immunosuppressive effect when previously activated with proinflammatory factors [29, 39, 40], MSCs were primed with 10 ng/mL of tumour necrosis factor-*α* (TNF*α*) and interferon-*γ* (INF*γ*). Both BM-MSCs and UCX, primed and naïve, suppressed T cell proliferation (Figure 3(a)). Priming with TNF*α* and IFN*γ* resulted in only a slight increase in the suppressive effect of both cell types. Interestingly, a significant increase in suppression of lymphocyte proliferation by naïve UCX when compared with naïve BM-MSCs was observed, suggesting that UCX are also naturally more immunosuppressive than BM-MSCs.

The induction of regulatory T cells (Tregs) is one possible mechanism for the suppression of the allogeneic T cell response. Therefore, the cells' ability to suppress the immune system through the induction of Tregs was also assessed (Figure 3(b)). Foxp3 remains the best marker to identify regulatory T cell population [41]. Therefore, in this study, we assessed Foxp3 expression in FACS-sorted CD3^+^CD4^+^ T cells to determine Treg formation. CD3^+^CD4^+^CD25^−^Foxp3^−^ T cells were incubated with TGF-*β*1 (10 ng/mL) or an irradiated non-MSC cell line (Molt4), irradiated BM-MSCs, and irradiated naïve and primed UCX. Results showed that incubation with TGF-*β*1 resulted in approximately 40% conversion to CD3^+^CD4^+^CD25^+^Foxp3^+^ cells. In the absence of exogenous TGF-*β*1, Treg conversion was also observed following incubation of the CD3^+^CD4^+^CD25^−^Foxp3^−^ T cells with irradiated BM-MSCs (16% ± 1%), primed BM-MSCs (14% ± 2%), UCX (19% ± 1%), and primed UCX (16% ± 4%). Treg conversion was not observed when CD3^+^CD4^+^CD25^−^Foxp3^−^ T cells were incubated with irradiated Molt4 cells. Consistent with the lymphocyte suppression assay (Figure 3(a)), these results showed that naïve UCX were modestly yet significantly more immunosuppressive than naïve BM-MSCs and that priming of UCX with TNF*α* and IFN*γ* did not significantly increase the cells ability to induce Treg conversion. These results suggest that UCX are strong inducers of Tregs and need no priming or activation for that effect.

Overall, these results indicate that UCX show superior immunosuppressive potential when compared to BM-MSCs through two different mechanisms: (1) inhibition of lymphocyte activation and proliferation and (2) induction of regulatory T cells. In addition, unlike what is known for BM-MSCs, UCX need no activation or priming to exert their immunosuppressive effect.

### 3.4. UCX Are Anti-Inflammatory* In Vivo* without the Need for Priming

**Figure 4:**
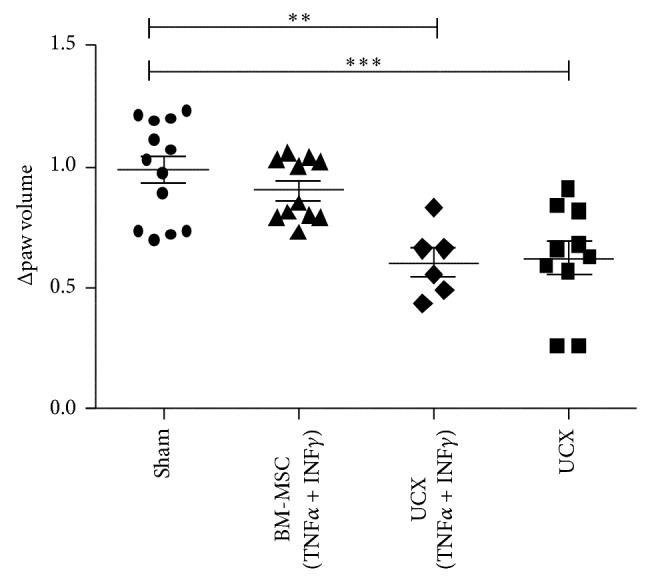
Effect of UCX in an acute inflammation model. Wistar rats (7 to 8 weeks old) were treated either with PBS vehicle (sham control), BM-MSCs (1.7 × 10^6^ cells), UCX (1.7 × 10^6^ cells), or UCX previously activated with 10 ng/mL of TNF*α* and IFN*γ* (1.7 × 10^6^ cells), 1 hour prior to challenge with *λ*-carrageenan in the right paw. Anti-inflammatory effect* in vivo* was assessed by measuring the paw volume at maximum peak time – 6 h, relative to the preinjection volume. Data is presented as mean ± s.e.m. and statistically significant differences are indicated by asterisks (nonparametric test Mann Whitney; ^∗^ *P* < 0.05, ^∗∗^ *P* < 0.01, and ^∗∗∗^ *P* < 0.001).

The* in vitro* data presented here suggests that priming is not necessary for UCX to exert immunomodulation. Using an acute carrageenan-induced arthritic (CIA) inflammatory model, these properties were further tested* in vivo*. This model is routinely used to test the efficacy of anti-inflammatory drugs locally injected in the rat hind paw, followed by an injection of carrageenan, which induces an acute inflammatory response. Upon carrageenan injection, the paw swells with inflammation peaking at around 6 hours, after which swelling starts to decrease to normal levels. In this study, 7- to 8-week-old Wistar rats were treated either with PBS vehicle (sham control), 1.7 × 10^6^ of BM-MSCs or UCX primed with 10 ng/mL of TNF*α* and IFN*γ*, or naïve UCX, 1 hour prior to challenge with *λ*-carrageenan in the right hind paw. Oedema was measured as the increase in paw volume (millilitres) after carrageenan injection (relative to the preinjection volume). Results showed that by 6 hours all MSCs reduced paw swelling, though naïve UCX showed the highest significant difference when compared to sham control (Figure 4). This data further confirms that priming UCX with TNF*α* and INF*γ* does not enhance the cells' immunomodulatory properties and that naïve UCX have a potent anti-inflammatory activity* in vivo*.

### 3.5. Comparison of Gene Expression Profile of UCX and BM-MSCs

**Figure 5:**
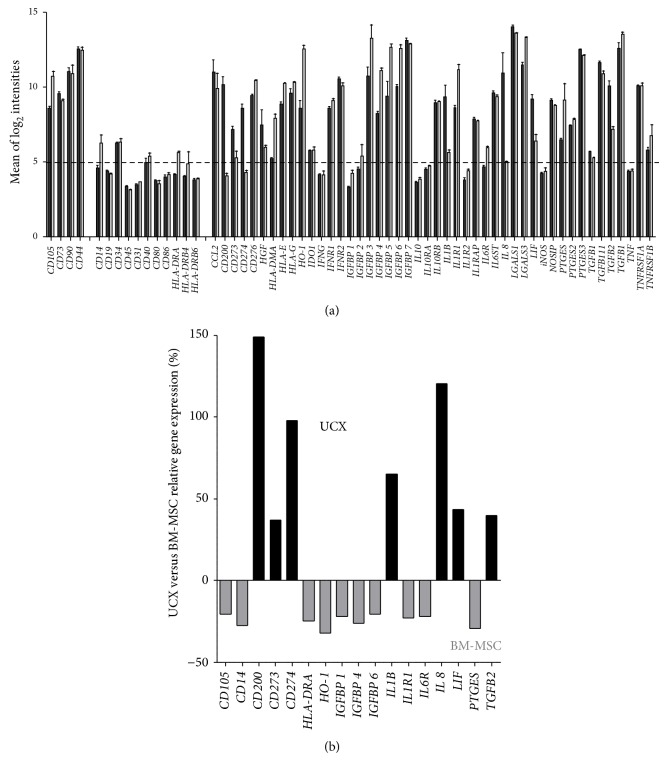
Comparison of UCX (black bars) and BM-MSCs (grey bars) transcriptome of immunomodulatory genes. (a) Relative gene expression of known MSC and immune response related genes in UCX (derived from 3 different umbilical cords) and BM-MSCs (2 different donors) are represented as mean ± s.e.m. Unbroken line was positioned on the average negative known markers of MSCs. Genes with relative expression values above this line were considered substantially expressed. (b) Genes where there was a relative expression significantly increased or decreased 20% or more.

In order to begin elucidating the mechanisms behind the functional differences observed between UCX and BM-MSCs, expression profiling was used to analyse genes described as being involved in immune responses, modulation and tolerance pathways, and the relative expression of core negative and core positive markers for MSCs. Affymetrix full genome expression analysis (Gene Chip HG_U133 Plus 2.0) was performed on 3 cultures of UCX derived from 3 distinct umbilical cords and on BM-MSCs from 2 different donors. All MSC cultures expressed high transcript levels for MSC markers* CD105*,* CD73*,* CD90*, and* CD44* and low levels for* CD19*,* CD34*,* C45*,* CD31*, and* HLA-DR* (Figure 5(a)), consistent with the flow cytometry results shown in Figure 1. In addition, results show that the mRNAs for the costimulatory molecules* CD80* and* CD86* were, as expected, not highly expressed. However, BM-MSCs seem to express higher levels of* CD14*, a marker for monocytes and macrophages.

**Figure 6:**
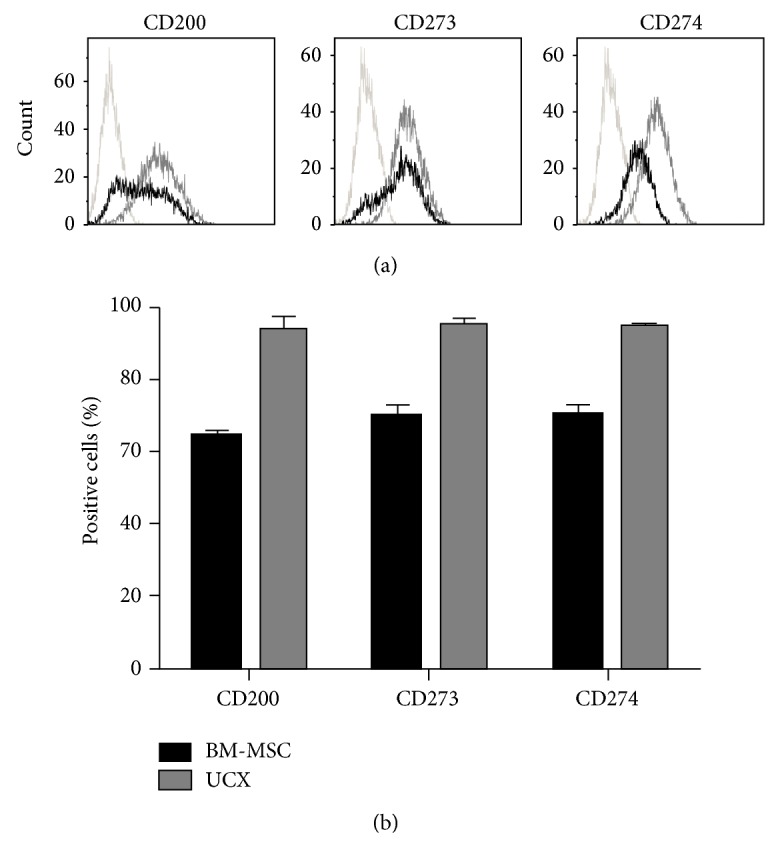
Flow cytometry surface expression studies of CD200, CD273, and CD274 in UCX and BM-MSCs. (a) Representative histograms of staining for the three surface proteins in UCX (grey lines) and BM-MSCs (black lines) and respective isotype controls. (b) Graphical representation of the percentage of cells positive for each surface protein from 3 different samples of each cell type (between passages 5 and 7) and presented as mean ± s.e.m. A higher percentage of UCX cells constitutively express CD200, CD73, and CD274 when compared to BM-MSCs.

Overall, the gene expression profile of UCX and BM-MSCs presented herein confirms their MSC character and their low immunogenicity and strongly supports an immunomodulatory potential. However, some differences were identified between the MSCs from these two sources.  Figure 5(b) depicts the genes for which the relative expression in UCX was significantly increased or decreased by 20% or more when compared to that in BM-MSCs. Results showed that the relative expression of* CD105*,* CD14*,* HLA-DRA*,* HO-1*,* IGFBP 1*,* IGFBP 4*, and* IGFBP 6*,* IL1R1*,* IL6R*, and* PTGES* transcripts was lower in UCX when compared to BM-MSCs. Conversely, the relative gene expression of* CD200*,* CD273*,* CD274*,* IL-1B*,* IL-8*,* LIF*, and* TGFB2* was significantly higher in UCX when compared to BM-MSCs. These differences may affect the immunogenicity and immunomodulatory properties of each cell type and may explain the enhanced immunosuppressive and anti-inflammatory effects observed in UCX. For that reason, the protein expression of the latter genes was studied in both cell types. Expression of CD200, CD273, and CD274 was analysed by flow cytometry in cells between passages 5 and 7, showing that all three surface proteins were highly expressed in UCX (>90%) while BM-MSCs expressed only 64.5%, 70%, and 71%, respectively (Figure 6).

**Figure 7:**
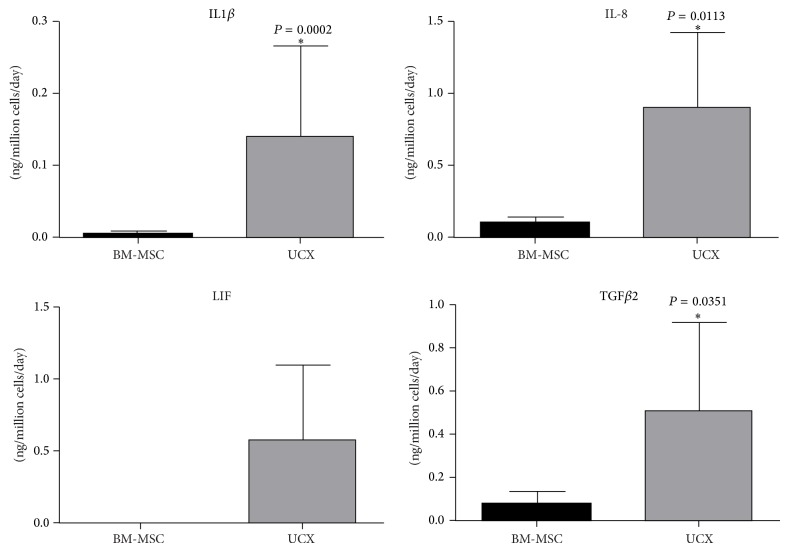
Quantification of IL-1*β*, IL-8, LIF, and TGF-*β*2 in conditioned media from UCX and BM-MSCs. Except for IL-8 which was quantified using FlowCytomix, all other secreted proteins were quantified by ELISA. Values are represented as mean ± s.e.m. and statistically significant differences are indicated by asterisks (unpaired *t*-test). No statistical analysis was performed in the case of LIF since this protein was not detected in conditioned media from BM-MSCs. All 4 secreted factors were detected in higher amounts in conditioned media obtained from UCX cells.

The secretion of soluble factors IL-1*β*, IL-8, LIF, and TGF*β*2 was studied by analysing conditioned media prepared from both cell types by FlowCytomix (IL-8) and by ELISA (IL-1*β*, LIF, and TGF*β*2) (Figure 7). Results showed that there was a significant increase in the expression of IL-1*β*, IL-8, LIF, and TGF*β*2 in UCX conditioned media. In fact, LIF was not even detectable in BM-MSC conditioned media. Altogether, these results confirm the gene expression data obtained. These 7 proteins are most probably coresponsible for the enhanced immunosuppressive and anti-inflammatory activities observed in UCX, compared with BM-MSCs.

## 4. Discussion

Though MSCs from different sources are often discussed as if they were one cell population, it is becoming widely accepted that there may be differences in their phenotype, including their immune regulatory properties [42, 43]. We find, for example, that the osteogenic potential of BM-MSCs is higher than that of umbilical cord tissue-derived cells and the reverse is observed for chondrogenic differentiation (Figure 1). Yet, despite low and inconsistent yields which decrease progressively with advancing donor age, BM-MSCs are still the most commonly used source of adult MSCs in clinical research with a frequency of colony-forming unit-fibroblast (CFU-F) of 1 : 35700 in the BM nucleated cells compared to 1 : 1609 in umbilical cord nucleated cells [44]. In addition to a lower frequency, BM-MSCs also seem to have lower proliferation rates and have limited expansion capability.

The main goal in the present work was to determine whether, beyond their expansion capabilities and good recovery rates, UCX also displayed properties that would make them more attractive for allogeneic cellular therapies. Specifically, UCX displayed a more beneficial immunogenic profile over BM-MSCs (Figure 2), as assessed by an allogeneic lymphocyte stimulation assay (MLR) using MSCs around passage 6. BM-MSCs, which are known to be nonimmunogenic, were observed to induce low levels of lymphocyte proliferation, most probably due to their advanced age in culture (p6). While passage 6 may induce senescence in BM-MSCs, UCX at this passage are far from showing any signs of ageing (data not shown). Hence, consistent with other studies [45], UCX did not elicit allogeneic responses* in vitro*. While the levels of* CD80*,* CD86*, and* CD40* gene expression are similar between the two sources of MSCs, there is an increased level of* HLA-DR* gene expression in BM-MSCs when compared to UCX (Figure 5(b)). This is consistent with our findings where UCX demonstrated lower immunogenicity when compared to BM-MSCs and with other reports [46] that showed similar results for human umbilical cord lining MSCs. This report also showed that umbilical cord lining MSCs have a stronger overall immunosuppressive potential when compared to BM-MSCs, which is in line with the findings presented here. Also, in another study [40], WJMSCs were shown to be more suppressive than BM-MSCs in MLR using phytohaemagglutinin-activated lymphocytes.

It has become widely assumed that, in order for MSCs to exert their immunomodulatory effect, they must be primed or previously activated [29, 39, 47]. Based on the results presented here, it is proposed that this is not the case for UCX in lymphocyte suppression, induction of Tregs, or* in vivo* anti-inflammatory effects. This observation is also supported by others [40] who found that priming with inflammatory stimuli can enhance the ability of BM-MSCs but not the ability of WJMSCs to suppress mitogen induced lymphoproliferation. Hence, UCX seem to be naturally more potent immunosuppressors and less immunogenic than BM-MSCs.

Further to their ability to modulate adaptive immunity, it was also shown that UCX can modulate innate immune responses* in vivo*. Here, we report that, in a model of acute inflammation, treatment with UCX resulted in reduced swelling and decreased inflammation in only 6 hours.

To identify potential mechanisms of action that could explain the observed functional differences, gene expression profiling was performed in both UCX and BM-MSCs and major differences were analysed.

Significant transcription expression differences were found between UCX and BM-MSC (Figure 5) suggesting that the immunomodulatory effect by both cell types may be regulated by different factors and pathways.* HO-1*, a potent immunosuppressive enzyme, and* PTGES* were found to be expressed substantially in BM-MSC indicating that these molecules may be more important in BM-MSC immunomodulation. Conversely, CD200, CD273, CD274, IL-1*β*, IL-8, LIF, and TGF-*β*2 were found to be substantially expressed in UCX and thus it is proposed that all or some of them may be responsible for the potent immunomodulatory capacities observed by UCX. CD200, LIF, and CD274 (or PD L1) play an essential role in conferring fetomaternal tolerance in an allogeneic pregnancy model, that is, in suppressing the maternal immune response to paternally inherited alloantigens [22, 48]. Hence, the functional increase in immunosuppressive capacity of UCX observed in this study (Figure 3) may be explained by the high expression of these genes. Interestingly, while priming of BM-MSCs leads to an increase in the percentage of cells that express CD200, the same was not observed in WJ-MSCs [27]. In our hands, >90% of naïve UCX express CD200 so there is very little room for improvement regarding the number of cells in the population that express CD200.

CD274 has been described by others to be present in 35 to 42% of UC-MSCs and upregulated to >95% in IFN*γ*-treated UC-MSCs [23]. In our hands, >90% of UCX express CD274 on their surface and this was only marginally increased when cells were treated with IFN*γ* (results not shown). These apparent differences may be due to different methods of isolation of MSCs from the umbilical cord tissue. More recently, it has been suggested that IFN*γ*-licensed BM-MSCs inhibit T cell effector function independent of IDO but through the ligands for PD1, CD273, and CD274 [49]. It is proposed that one of the reasons why UCX show enhanced immunosuppressive capabilities, without the need for priming, is their high constitutive expression levels of these ligands (>90%), which were found to be significantly higher when compared to (unprimed) BM-MSCs.

LIF, which was originally shown to be involved in BM-MSC modulation of T-lymphocytes [22], has since been described to be implicated in the expansion of regulatory T cells [50] and, consistent with our studies, it was found to be expressed in higher levels in WJ-MSCs when compared to BM and adipose tissue-derived MSCs (AT-MSCs).

The elevated expression of IL-8, as well as IL-1*β*, by UCX is consistent with other studies comparing BM-MSCs with umbilical cord vein and Wharton's jelly derived MSCs [51, 52]. High expression of proinflammatory cytokines may be counterintuitive in the context of anti-inflammatory properties. However, it is still not clear how each MSC type exerts its immunomodulatory function. The evidence presented here and elsewhere [34] undisputedly shows that, despite their high levels of IL-8 and IL-1*β*, UCX are immunosuppressive and anti-inflammatory. The high levels of these factors may speak more of other therapeutic effects by these cells, such as revascularization and commitment to angiogenesis ([51] and our own unpublished data). TGF-*β*2, which was also upregulated in UCX, may also play a role, though the MSCs' ability to generate Tregs was found only to correlate with the amount of TGF-*β*1 (not TGF-*β*2) secreted by the MSCs (data not shown). Overall, the results presented in this work suggest that UCX are strong candidates for cellular therapy, not only in autologous and HLA-matched heterologous grafts, but also in allogeneic immunotherapies. Importantly, the results show that not all MSCs behave the same and, depending on the source of the cells, MSCs may be more or less immunogenic and more or less potent and even, potentially, may show therapeutic benefit for different applications in the clinic. Generalisations on the biology and clinical application of MSCs based on studies from a single source of the cells should therefore be avoided.
